# Discovery of the first unconventional myosin: *Acanthamoeba* myosin-I

**DOI:** 10.3389/fphys.2023.1324623

**Published:** 2023-11-17

**Authors:** Thomas D. Pollard, Edward D. Korn

**Affiliations:** ^1^ Department of Molecular Cellular and Developmental Biology, Yale University, New Haven, CT, United States; ^2^ Department of Molecular and Cell Biology, University of California, Berkeley, CA, United States; ^3^ Scientist Emeritus, Laboratory of Cell Biology, National Heart, Lung, and Blood Institute, National Institutes of Health, Bethesda, MD, United States

**Keywords:** actin, myosin, myosin I, unconventional myosin, *Acanthamoeba*

## Abstract

Having characterized actin from *Acanthamoeba castellanii* (Weihing and Korn, Biochemistry, 1971, 10, 590–600) and knowing that myosin had been isolated from the slime mold *Physarum* (Hatano and Tazawa, Biochim. Biophys. Acta, 1968, 154, 507–519; Adelman and Taylor, Biochemistry, 1969, 8, 4976–4988), we set out in 1969 to find myosin in *Acanthamoeba*. We used K-EDTA-ATPase activity to assay myosin, because it is a unique feature of muscle myosins. After slightly less than 3 years, we purified a K-EDTA ATPase that interacted with actin. Actin filaments stimulated the Mg-ATPase activity of the crude enzyme, but this was lost with further purification. Recombining fractions from the column where this activity was lost revealed a “cofactor” that allowed actin filaments to stimulate the Mg-ATPase of the purified enzyme. The small size of the heavy chain and physical properties of the purified myosin were unprecedented, so many were skeptical, assuming that our myosin was a proteolytic fragment of a larger myosin similar to muscle or *Physarum* myosin. Subsequently our laboratories confirmed that *Acanthamoeba* myosin-I is a novel unconventional myosin that interacts with membrane lipids (Adams and Pollard, Nature, 1989, 340 (6234), 565–568) and that the cofactor is a myosin heavy chain kinase (Maruta and Korn, J. Biol. Chem., 1977, 252, 8329–8332). Phylogenetic analysis (Odronitz and Kollmar, Genome Biology, 2007, 8, R196) later established that class I myosin was the first myosin to appear during the evolution of eukaryotes.

## 1 Introduction

Today it must be difficult for a person entering scientific research to appreciate how little was known in 1969 about the molecular basis of cellular movements. Furthermore, the tools available to study this fundamental life process were very limited. These were the days before SDS-gel electrophoresis, methods to determine gene sequences, personal computers, the internet, fluorescence microscopy for cell biology and digital cameras. Some investigators were even skeptical that anything could be learned about cellular movements using biochemical methods.

Nevertheless, pioneering work by Sadashi Hatano and Fumio Oosawa (Hatano and Oosawa, 1966; Hatano and Tazawa, 1968) in Japan and Mark Adelman and Edwin Taylor (Adelman and Taylor, 1969b; Adelman and Taylor, 1969b) in Chicago revealed by purification and characterization that the acellular slime mold *Physarum* has proteins similar to muscle actin and myosin. *Physarum* myosin is a large protein with a heavy chain that resembles the muscle myosin heavy chain in size.

Bob Weihing initiated work on motility proteins in the Korn lab at NIH by purifying actin from *Acanthamoeba* (Weihing and Korn, 1971). When Pollard joined the Korn lab in the summer of 1969, he and Weihing showed that the heavy meromyosin fragment of muscle myosin forms arrowhead-shaped complexes on amoeba actin filaments (Pollard et al., 1970), as first described for muscle actin filaments by Hugh Huxley (Huxley, 1963). Pollard and Weihing also used a method described by Hal Ishikawa in Howard Holtzer’s lab (Ishikawa et al., 1969) to decorate actin filaments in the cytoplasm of extracted *Acanthamoeba* cells with myosin (Pollard et al., 1970).

### 1.1 Discovery of *Acanthamoeba* myosin-I

We set out to find myosin in *Acanthamoeba*, fully expecting a large protein like muscle and *Physarum* myosins. On the advice of Evan Eisenberg, a postdoc with Wayne Kielley in our Department, we used an ATPase assay with EDTA and a high concentration of KCl to monitor the purification process. Myosin was the only known ATPase with high activity under these non-physiological conditions. After we made some progress with the purification, we added a parallel assay with actin filaments to stimulate the Mg-ATPase activity, a second novel feature of known myosins.

**FIGURE 1:**
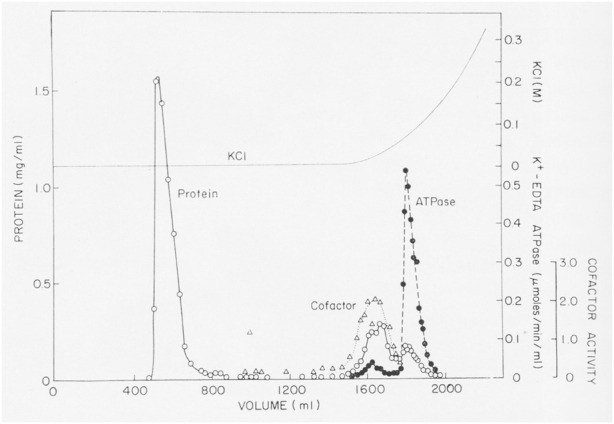
Purification of *Acanthamoeba* myosin-I by adsorption to and elution from an agarose gel filtration column. About 40 mL of partially purified myosin-I were applied to a 5 × 75 cm column of Bio-Rad A1.5m (8% agarose) and eluted with 0.2 mM ATP, 1 mM DTT, 2 mM Tris chloride (pH 7.6). No K-EDTA-ATPase activity eluted until the column was eluted with a gradient of KCl Figure 3 in Pollard and Korn (1973b).

Over a period of almost 3 years and more than 70 separate experiments, we purified an enzyme with K-EDTA-ATPase activity, but the protein was much smaller than muscle myosin. The crucial step in the purification was discovered accidently, when we tried to purify a crude fraction with K-EDTA-ATPase activity by gel filtration on a Bio-Rad A1.5m gel filtration column in a low ionic strength buffer (Figure 1). Most of the applied protein flowed through the column, but disappointingly, no ATPase activity eluted. In desperation, Pollard eluted the column with a gradient of KCl. Surprisingly, fractions containing protein emerged from the column, cleanly separating highly purified myosin from contaminants. Apparently, this batch of A1.5m had enough charged groups to act as a very weak ion exchange column. (Later batches of Bio-Rad A1.5m did not have this “magical” activity.) Agarose adsorption became the key step in the purification of *Acanthamoeba* myosin, allowing us to isolate about 3 mg from 600 g of cells using anion exchange chromatography, ammonium sulfate precipitation, agarose adsorption chromatography and hydroxyapatite chromatography (Pollard and Korn, 1973a).

**FIGURE 2:**
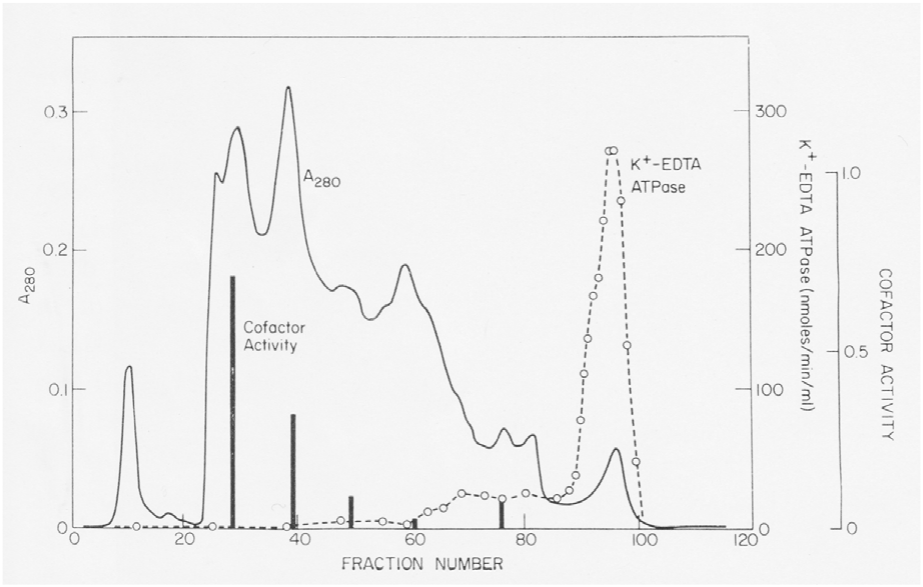
Discovery of cofactor protein. Crude *Acanthamoeba* myosin was purified by ion exchange on DEAE-cellulose, fractionation with ammonium sulfate and gel filtration on a column of 8% agarose beads in 500 mM KCl. Crude myosin was run through a column of hydroxyapatite in 500 mM KCl and eluted with a gradient of potassium phosphate. K-EDTA ATPase activity eluted at high phosphate concentrations, but these fractions lacked Mg-ATPase activity stimulated by actin filaments. However, enzymatically inactive fractions that eluted earlier stimulated the actin-activated Mg-ATPase activity of *Acanthamoeba* myosin (black bars). This was the “cofactor” activity Figure 2 in Pollard and Korn (1973b).

Actin filaments stimulated the Mg-ATPase activity of partially pure *Acanthamoeba* myosin, but this activity was lost with further purification. Sulfhydryl oxidation may have a similar effect on muscle myosin, so we assumed that our protein was damaged during purification. Fortunately, Pollard met Edwin Taylor on a trip to the University of Chicago. Taylor made a crucial suggestion: recombine column fractions without ATPase activity with the fraction having K-EDTA-ATPase activity and assay for Mg-ATPase activity with actin filaments. Upon returning to Bethesda, Pollard dialyzed phosphate out of fractions from a hydroxyapatite column where the fractions with K-EDTA ATPase activity lost their actin-activated Mg-ATPase activity. Pollard was delighted to discover other fractions from the column without ATPase activity stimulated the actin-activated Mg-ATPase activity of the myosin (Figure 2). No one else was in the lab on Saturday, so he telephoned Korn at home with the welcome news. We called this activity the “cofactor” protein and partially purified it (Pollard and Korn, 1973b). Subsequently, we learned that agarose adsorption also separated cofactor protein from *Acanthamoeba* myosin (Figure 1).

Purified *Acanthamoeba* myosin consisted of a single heavy chain of about 140 kDa with a single ATPase head and two light chains. It bound to actin filaments in the absence of ATP but not in the presence of ATP. However, it differed fundamentally from myosins purified from muscle, *Physarum* and animal nonmuscle cells. The Stokes’ radius was consistent with a globular protein of about 180 kDa. Despite its small size, *Acanthamoeb*a myosin appeared in electron micrographs to crosslink pairs of actin filaments. In contrast, muscle myosin has two ATPase heads and a long tail formed by a coiled-coil of the two 200 kDa heavy chains. *Acanthamoeba* myosin was soluble at low ionic strength, conditions where myosins from skeletal muscle (Huxley, 1963), *Physarum* (Nachmias, 1972), human platelets (Adelstein et al., 1971) and fibroblasts (Adelstein et al., 1972) form bipolar filaments.

These properties led many in the myosin field to conclude that the *Acanthamoeba* myosin was a degradation product of a myosin similar to muscle myosin. In 1864 (yes, 1864), the German physiologist W. Kühne extracted a salt-soluble protein from muscle that he called “myosin” (from the Greek word “mus” for “muscle” (Kühne, 1864). Every myosin isolated from any organism before 1973 resembled muscle myosin, so it was reasonable to suspect that the smaller myosin we isolated from *Acanthamoeba* was a proteolytic fragment of a typical myosin.

Furthermore, *Acanthamoeba* myosin was the first myosin shown to require a cofactor protein for actin-activated ATPase activity. Additional experiments produced fractions enriched in a 97 kDa protein with cofactor activity (Pollard and Korn, 1973b). The Mg-ATPase activity of *Acanthamoeba* myosin depended in a biphasic fashion on the concentrations of actin filaments with a unique peak of activity at low actin concentrations followed by a hyperbolic dependence on higher concentration of actin as seen for other myosins. We suggested that the low actin-activated ATPase activity of purified platelet myosin (Adelstein et al., 1971) might be due to a missing cofactor protein.

### 1.2 Advances in understanding *Acanthamoeba* myosin-I

Subsequent work by the Korn laboratory found that *Acanthamoeba* contains 3 myosin-I isoforms, (myosin-IA, myosin-IB and myosin-IC) none of which forms filaments (Maruta et al., 1979). Both of our laboratories independently purified from *Acanthamoeba* a myosin similar to muscle myosin (Maruta and Korn, 1977a; Pollard et al., 1978). Electron microscopy showed that this myosin has two ATPase heads, a 90 nm long coiled-coil tail (much shorter than muscle myosin 150 nm) and forms short bipolar filaments (Pollard et al., 1978). John Hammer provided the definitive evidence that the small and large *Acanthamoeba* myosins are different gene products by isolating genomic DNA and sequencing regions of the heavy chains (Hammer et al., 1986a; Hammer et al., 1986b; Jung et al., 1987).

The small amoeba myosins with one heavy chain and one ATPase head became “myosin-I” and the larger myosin with two heavy chains and two ATPase heads became “myosin-II.” Myosin-II has long been considered to be “conventional” myosin, and all others, beginning with *Acanthamoeba* myosin-I, are now called “unconventional” myosins. Additional classes of myosin were numbered in order of their discoveries.

**FIGURE 3:**
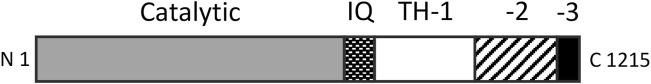
Domain organization of *Acanthamoeba* myosin-IA. The catalytic domain has ATPase activity and binds actin filaments in the absence of ATP. The three IQ domains bind light chains. The basic tail homology 1 (TH-1) domain binds acidic membrane lipids and actin filaments. The TH-2 domain binds actin filaments and folds back to interact with the TH-1 domain. TH-3 is an SH3 domain. The organization of myosin-IB is similar, while myosin-IC has the SH3 domain within the TH2 domain. Redrawn from (Lee et al., 1999).

**FIGURE 4:**
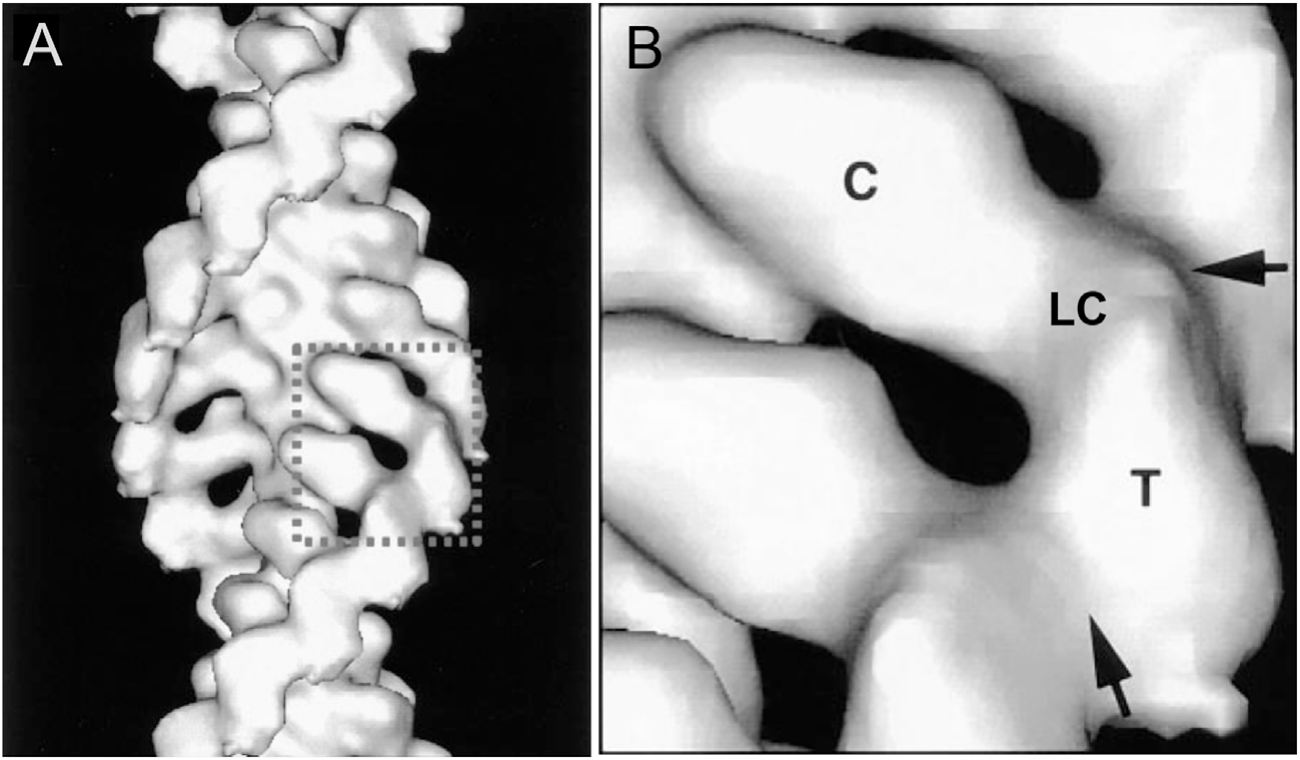
Three dimensional reconstruction at 18 Å resolution of cryo-electron micrographs of *Acanthamoeba* myosin-1B bound to actin filaments. **(A)**, overview; **(B)**, detailed view of one molecule. The catalytic domain (C) of one myosin-I is bound to each actin subunit. A narrow region with the single light chain (LC) links the catalytic domain to the tail domains (T). Arrows mark contacts between the tails. Modified from Figure 4 (Jontes et al., 1998).

Over a period of 20 years, our labs determined the primary structures of the three *Acanthamoeba* myosin-Is and a light chain. The domain structure of myosin-IA (Figure 3) (Lee et al., 1999) is typical of the other two myosin-Is (Brzeska et al., 1988; Lee et al., 1999). The large, N-terminal catalytic domain is homologous to the heads of other myosins. The next domain has binding sites for three light chains. Myosin-IB and myosin-IC have single light chains (Liu et al., 2000). The tail has three “tail homology” domains that fold independently: a basic TH1 domain; a TH2 or GPA-rich (glycine proline alanine) domain; and a TH3/SH3 domain, which is located at the end of the heavy chains of myosin-IA and myosin-IB or in the middle of the TH2 domain in myosin-IC (Liu et al., 2000). The TH1 and TH2 domains bind to each other and bind independently to actin filaments in the presence or absence of ATP (Lee et al., 1999). The hydrodynamic properties of isolated tail domains and their size and shape in reconstructions from electron micrographs (Figure 4) suggest that the TH2/TH3 domains fold back on the TH1 domain (Lee et al., 1999).

Richard Adams discovered that *Acanthamoeba* myosin-I binds membranes (Adams and Pollard, 1989) and transports membrane vesicles along actin filaments (Adams and Pollard, 1986). The TH1 domain in the proximal part of the myosin-I tail binds acidic phosphoglycerides (Doberstein and Pollard, 1992), allowing myosin-I to move actin filaments over the surface of supported lipid bilayers (Zot et al., 1992).

A pre-steady state kinetic analysis by Michael Ostap (Pollard and Ostap, 1996) revealed that the actomyosin ATPase cycle of *Acanthamoeba* myosin-I is similar to muscle myosin with a small duty cycle. Thus, multiple myosin-I molecules must work cooperatively to move a vesicle on an actin filament. The peak of steady state Mg-ATPase activity at low actin filament concentrations was explained by myosin-I crosslinking actin filaments through interactions of the tail with one filament and the cycling head with another filament (Lynch et al., 1986; Brzeska et al., 1988).

Three dimensional reconstructions of *Acanthamoeba* myosin-IB (Jontes et al., 1998) and myosin-IC (Carragher et al., 1998) bound to actin filaments showed that the head binds actin filaments like muscle myosin and a short tail extends from the head.

The physiological functions of *Acanthamoeba* myosin-I’s are less well understood than their biochemical properties. However fluorescent antibody localization in fixed cells (Baines et al., 1992; Baines et al., 1995), loading fluorescent antibodies into live cells (Ostap et al., 2003) and transient expression of EGFP-myosin-IC in live cells (Kong and Pollard, 2002) implicated myosin-I isoforms in macropinocytosis, phagocytosis, heterophagy and contractile vacuole function. Transient concentration of myosin-IC around contractile vacuoles required both the head and the SH3 domain in the tail (Kong and Pollard, 2002). Loading *Acanthamoeba* cells with inhibitory antibodies to myosin-IC (but not myosin-IB) disrupted contractile vacuole function, and cells swelled and lysed in hypotonic media (Doberstein et al., 1993).

Hiroshi Maruta purified the cofactor protein and discovered that it is a myosin heavy chain kinase (Maruta and Korn, 1977b) that phosphorylates a single serine (Hammer et al., 1983). Extensive additional work in the Korn lab characterized the heavy chain kinase and the phosphorylation sites (Brzeska et al., 1989).

### 1.3 Expansion of knowledge about myosin-Is

Myosin-I was discovered a second time as the physical link between the bundle of actin filaments inside microvilli of the intestinal brush border and the surrounding plasma membrane. This connection was first recognized in electron micrographs by Mooseker and Tilney (Mooseker and Tilney, 1975). Subsequently the protein was studied biochemically (Matsudaira and Burgess, 1979) and shown to be myosin-I by purification (Collins and Borysenko, 1984). Its primary structure (Hoshimaru and Nakanishi, 1987; Garcia et al., 1989) established that brush border myosin is a myosin-I with three calmodulin light chains and a lipid binding tail.

*Dictyostelium* amoebae were also found to contain a Class I myosin (Coté et al., 1985), and further work discovered a total of eight Class I myosins (IA-IH) (Falk et al., 2003). Some are homologous with *Acanthamoeba* myosin Is, including domain organization and membrane binding properties.

Research by many laboratories over the past 30 years revealed a remarkably wide range of physiological functions of myosin-I, particularly in animals [reviewed by (McIntosh and Ostap, 2016; Pernier and Schauer, 2022)]. For example, the eight isoforms of human myosin-I have specific functions revealed by combinations of genetic deletion, mRNA depletion, over expression, localization and *in vitro* reconstitution. A pioneering example implicated myosin-IC in the adaptation of sensory hair cells in the ear (Gillespie et al., 1993). Other functions include determining the left-right asymmetry of cells and organisms, delivering vesicles with the glucose carrier GLUT4 to the plasma membrane of muscle and fat cells in response to insulin, organizing cortical actin filaments, generating membrane tension and stabilizing focal adhesions. Decades of additional research are needed to understand how each myosin-I isoform is adapted to its physiological functions.

### 1.4 Gene sequences of thousands of myosins explain their diversity and evolution

Further research extended the classes of unconventional myosins. Myosin-III was discovered by genetic analysis of mutants affecting photoreceptors in flies (Montell and Rubin, 1988). It has an N-terminal kinase domain, followed by the catalytic domain and a tail that binds calmodulin. Horowitz and Hammer (Horowitz and Hammer, 1990) discovered myosin-IV by cloning the gene from *Acanthamoeba.* The gene encodes a myosin catalytic domain and a tail of 792 residues that differs from all other myosins except for the presence of a C-terminal SH3 domain. The distribution of myosin-IV is limited to *Acanthamoeba* and some unicellular organisms widely separated from amoebae on the phylogenetic tree (Kollmar and Mühlhausen, 2017). Myosin-V was discovered as a cell cycle mutant in budding yeast that encodes a protein required for vesicle transport into the bud (Johnston et al., 1991) and was isolated independently from chicken brain (Espindola et al., 1992). Thus by 1992 (19 years after discovery of the *Acanthamoeba* unconventional myosins) a total of only 14 unconventional myosins were identified forming five different classes of unconventional myosins.

The full extent of the myosin gene family was ultimately revealed by genome sequences of thousands of species spread across the phylogenic tree. By 2017 the inventory included 7,852 myosin genes from 929 organisms, divided into 79 classes (Kollmar and Mühlhausen, 2017).

**FIGURE 5:**
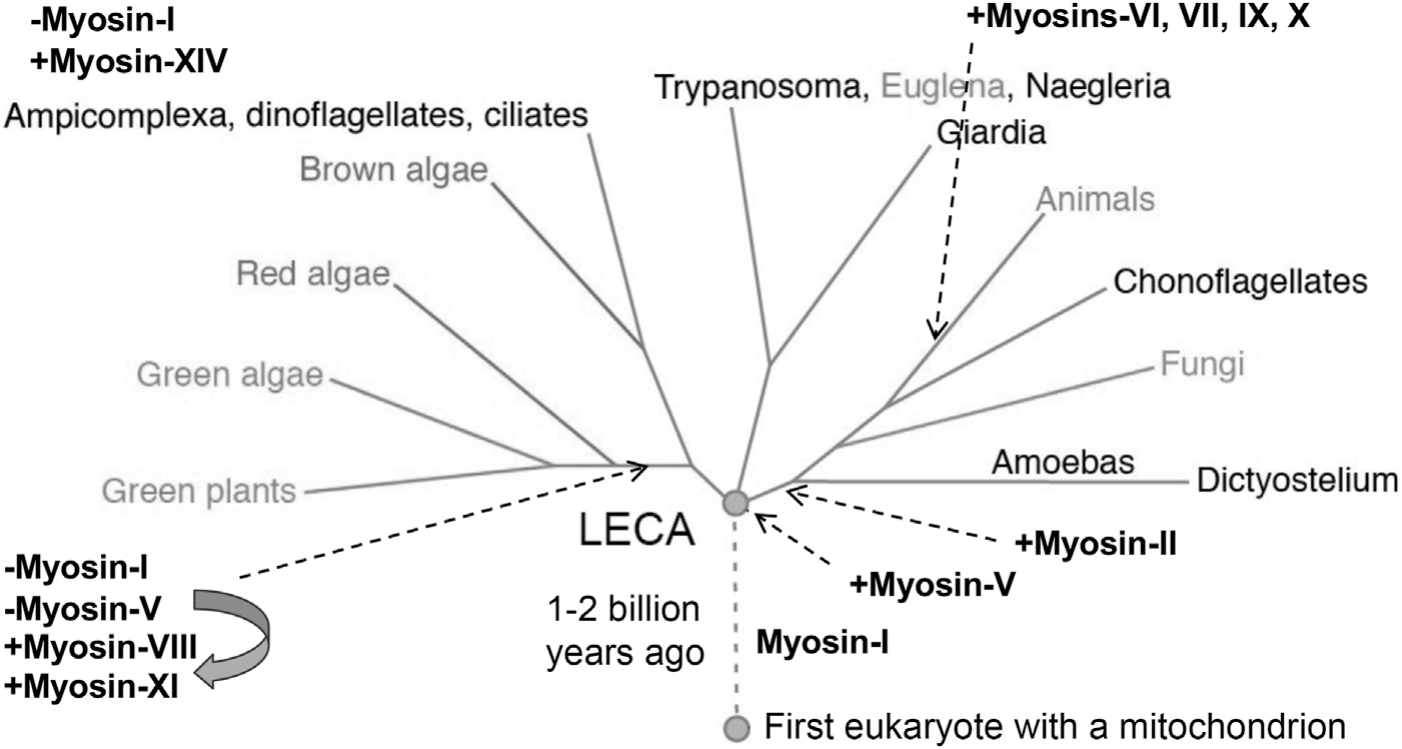
Phylogenetic tree showing the evolution of myosin genes beginning with the appearance of myosin-I more than 1 billion years ago between the time of the first eukaryote and the last eukaryotic common ancestor (LECA). Gene duplication closer to the time of LECA produced a second myosin related to myosin-V, which gave rise to all other myosin genes. Eukaryotes branching from LECA inherited these ancient myosin genes, but algae and plants lost the gene for myosin-I and the myosin-V gene evolved into genes for myosins-VIII and XI. Myosin-II appeared in the branch leading to animals, fungi and amoebas. Myosins-VI, VII, IX and X are confined to animals. Myosin-XIV replaced myosin-I in *Ampicomplexa*. [Positions of myosins are based on (Odronitz and Kollmar 2007)].

The first myosin gene to appear during evolution was for a class I myosin (Figure 5). Before the last eukaryotic common ancestor (LECA), the myosin-I gene duplicated and diverged to form a second class of myosin gene that became the precursor for all other classes of myosins. Further gene duplication and divergence produced genes for many new classes of myosin, such as myosin-II in the branch with amoebas, fungi and animals. Myosin genes have been lost many times and a few eukaryotes have none: the red algae *Cyanidioschyzon merolae*, *Porphyridium purpureum* and *Chondrus crispus*; as well as the protozoa *Giardia lamblia*, *Trichomonas vaginalis* and *Spironucleus salmonicida*.

### 1.5 Myosins and human disease

The discovery of unconventional myosins has had two big impacts. First, as noted by Edwin Taylor when he accepted the E. B. Wilson Medal from the American Society for Cell Biology (Taylor, 2001), it “…was the beginning of the end of the isolation of the muscle community from the rest of cell biology.” Second, continued research on unconventional myosins revealed that mutations of myosin genes predispose to many human diseases. A PubMed search for “myosins and human diseases” finds 6,317 papers, many on unconventional myosins. For example, deafness in humans is associated with mutations of heavy chain genes in Classes 1, 2, 3, 6, and 15; hearing and vision loss with mutation of Class 7 heavy chain genes; cardiac myopathies with mutations of Classes 2 and 6 heavy chain genes; platelet anomalies with mutation of Class 2 heavy chain genes; and hypopigmentosis and microvillus inclusion disease with mutation of Class 5 heavy chain genes (reviewed (Coluccio, 2020)). Thus, basic research on cellular movements opened the door for work required to understand human diseases.
